# Representation of non-coding RNA-mediated regulation of gene expression using the Gene Ontology

**DOI:** 10.1080/15476286.2024.2408523

**Published:** 2024-10-07

**Authors:** Giulia Antonazzo, Pascale Gaudet, Ruth C. Lovering, Helen Attrill

**Affiliations:** a Department of Physiology, Development and Neuroscience, University of Cambridge, Cambridge, UK; b SIB Swiss Institute of Bioinformatics, Swiss-Prot Group, Geneva, Switzerland; c Functional Gene Annotation, Institute of Cardiovascular Science, University College London, London, UK

**Keywords:** Gene Ontology, non-coding RNA, bioinformatics, biocuration, gene silencing, guidelines

## Abstract

Regulatory non-coding RNAs (ncRNAs) are increasingly recognized as integral to the control of biological processes. This is often through the targeted regulation of mRNA expression, but this is by no means the only mechanism through which regulatory ncRNAs act. The Gene Ontology (GO) has long been used for the systematic annotation of protein-coding and ncRNA gene function, but rapid progress in the understanding of ncRNAs meant that the ontology needed to be revised to accurately reflect current knowledge. Here, a targeted effort to revise GO terms used for the annotation of regulatory ncRNAs is described, focusing on microRNAs (miRNAs), long non-coding RNAs (lncRNAs), small interfering RNAs (siRNAs) and PIWI-interacting RNAs (piRNAs). This paper provides guidance to biocurators annotating ncRNA-mediated processes using the GO and serves as background for researchers wishing to make use of the GO in their studies of ncRNAs and the biological processes they regulate.

## Introduction

Non-coding RNAs (ncRNAs) have emerged as important regulators of biological processes, particularly through their role in controlling gene expression [1]. MicroRNAs (miRNAs), small interfering RNA (siRNAs) and P-element induced wimpy testis (PIWI)-interacting RNAs (piRNAs) can act post-transcriptionally by binding target mRNAs in the cytosol to suppress their translation [2–4]. Additionally, siRNAs and piRNAs can act in the nucleus by binding nascent transcripts to direct the genomic deposition of repressive epigenetic marks and suppress gene transcription [2,3]. These small regulatory RNAs share a common modality: the association with Argonaute family proteins (defined by the presence of PAZ and PIWI domains) to form an RNA-induced silencing complex (RISC)/RISC-related complex in which they act as sequence-specific guides to target RNAs [5,6]. Long non-coding RNAs (lncRNAs) are less easy to pigeonhole. While some lncRNAs have roles that overlap with those of the small regulatory RNAs, i.e., the post-transcriptional or epigenetic regulation of gene expression, they can also act in ways more commonly associated with proteins, such as scaffolding molecular assemblies in chromatin organization or nucleating phase-separation to drive the formation of non-membrane bound organelles [7]. There is a growing interest in lncRNAs, particularly as they have been shown to have a role in many diseases [8]. To date, the LncRNA and Disease Database (http://www.rnanut.net/lncrnadisease/) has curated 1,760 unique lncRNAs that have a causal link to disease [9]. For example, the aberrant expression of the lncRNA HOTAIR in non-small cell lung cancer is correlated with poor prognosis [10]. HOTAIR can base-pair with and suppress the activity of miR-149-5p [10] and miR-217-5p [11], resulting in the over-expression of the miRNA-targets HNRNPA1 and DACH1, respectively, thereby promoting proliferation and migration of lung cancer cells. As this example illustrates, ncRNAs can be part of complex regulatory networks that, if disrupted, can have major consequences. It therefore follows that having a computational base to model such networks is important to the interpretation of disease-associated data [12].

The Gene Ontology (GO) is a highly structured and systemized computational framework for describing the functional role of gene products through the use of standardized classification terms (GO terms) from three interlinked ontologies: biological process (BP), molecular function (MF) and cellular component (CC) [13,14]. MF terms are used to describe the detailed mechanism by which an individual gene product performs its role. The MF domain of the GO has been expanded in recent years to more precisely describe activities with two major aims: to better delineate how each gene product enacts its role in a larger biological program and to capture the effects on molecular targets of these activities [15]. For example, more terms have been created under the ‘*molecular adaptor activity*’ (GO:0060090) branch, such as ‘*histone reader activity*’ (GO:0140566) and ‘*molecular condensate scaffold activit*y’ (GO:0140693), which allow curators to capture binding activity of a gene product that brings other molecules together for a specific purpose and to not have to resort to less informative MF terms, such as ‘*protein binding*’ (GO:0005515). BP terms describe the programs requiring the coordinated action of multiple gene products to achieve a biological goal. The scope of the BP aspect of GO is broad in its range, encompassing cellular processes, such as DNA replication and metabolic pathways, to processes that involve intercellular coordination, from cell–cell signalling to the formation of anatomical structures. CC terms are used to describe where individual gene products perform their MF, such as a subcellular compartment, e.g., *‘nucleus’* (GO:0005634), or protein complexes to which they belong, e.g., *‘RISC complex*’ (GO:0016442). GO annotations can carry more information in the form of annotation extensions [16], these are additional data that are appended to the annotation that can give contextual information, for example, where an activity is carried out (e.g. nucleus) or the identifier (ID) of the target.

GO annotations are heavily weighted towards protein-coding genes, which reflects the historical focus on coding genes and their wide-ranging functions, and the relatively recent interest in ncRNAs. Since then, the understanding of miRNAs and other ncRNAs has progressed substantially, with more than 20,000 papers on ncRNAs now being published yearly [17]. In 2016, guidelines for the GO annotation of miRNAs were established to aid and encourage biocuration of this important class of gene products [18]. With the concerted efforts on miRNAs, 5,895 manual annotations to human miRNAs had been made by 2022. However, in contrast, by the same year only 38 manual annotations had been made to human lncRNAs, suggesting a substantial deficiency in curation. To begin to address the gap between the knowledge in research literature and the representation of regulatory ncRNAs in the GO, we have introduced more precise GO terms to describe the molecular functions of ncRNAs and the biological processes in which they are involved. We have expanded the focus beyond miRNA to better capture processes involving other types of regulatory ncRNAs: piRNAs, siRNAs and lncRNAs. Here we introduce these updates, give specific examples of annotation and present the data available to GO users. We expect that the present paper will serve both as a guide for biocurators to produce consistent annotation of ncRNAs and for users to facilitate the application of this resource in research projects.

## Results

### Revision of the GO to represent regulatory ncRNA biology

The GO was reviewed by a small group of curators and an ontologist in regular meetings. Specific GO MF and BP terms have been created or existing terms changed (such as changing ontological relations, names, definitions) to best represent the mechanism by which ncRNAs act and the processes they are part of. For a list of the major revisions to GO terms see Table S1. The sections below describe these terms and how they should be used in annotation. Additional links to curation guidelines and resources listed in the text are given in Table S2. All specific genes/gene products and their IDs used as examples in the text are listed in the Table S3.

### GO representation of post-transcriptional gene silencing by miRNAs, siRNA and piRNAs

Gene silencing by ncRNAs at the level of post-transcriptional targeting of mRNAs in the cytosol is one of the most well studied areas of ncRNA biology. This is primarily achieved by base-pairing with mRNAs to decrease their availability for translation by ribosomes. This activity is captured using a MF term in the *‘nucleic acid binding’* (GO:0003676) branch of the GO, ‘*mRNA base-pairing translational repressor activity’* (GO:1903231) (Figure S1A). The target of regulation can be specified using the annotation extension field with the relation ‘has_input’ to specify the target gene or gene product.

**Figure 1.**
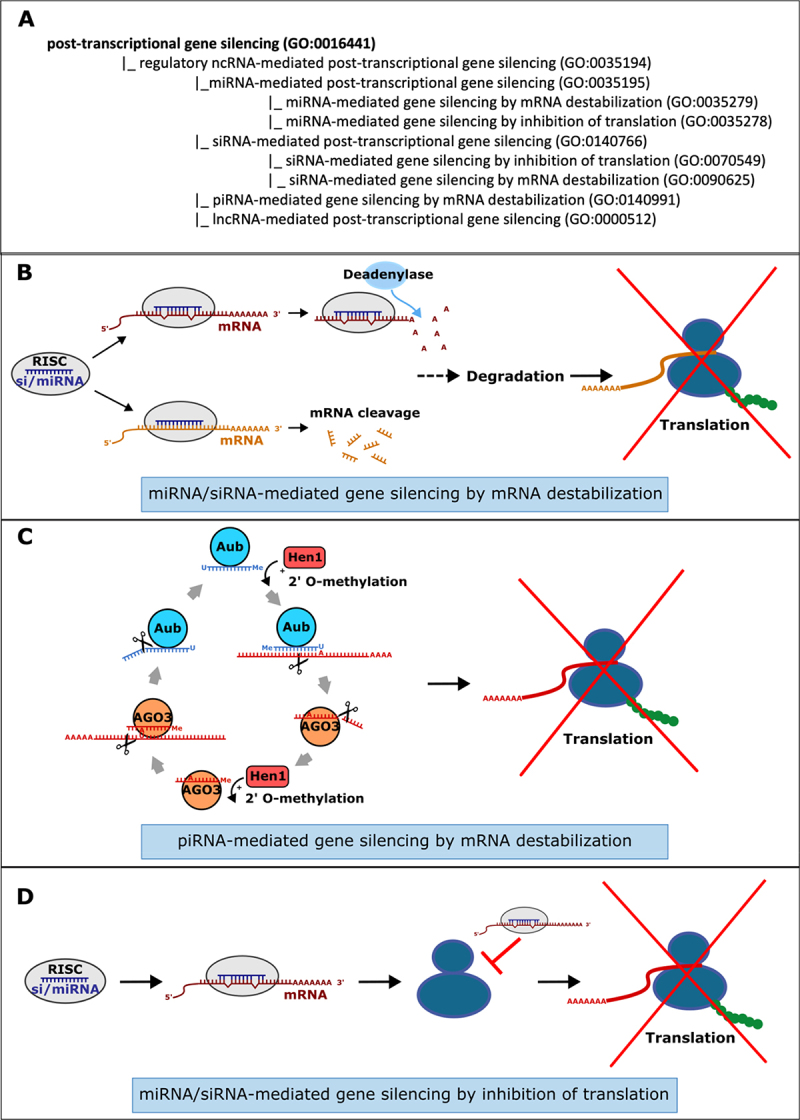
Regulatory ncRNAs can mediate PTGS. (A) Illustration of the GO terms hierarchy describing ncRNA-mediated PTGS. An expanded view provided in Figure S1C shows these terms in the context of the *‘regulation of gene expression’* (GO:0010468) branch of the GO. (B) PTSG mediated mi/siRNA gene silencing by mRNA destabilization. Imperfect complementarity with the guide RNA often leads to degradation after deadenylation of the mRNA target (top). Degradation through endonucleolytic cleavage of the mRNA in the RISC occurs when there is a near-perfect sequence complementarity with the guide RNA, as commonly happens in plants (bottom). (C) Link between secondary piRNA processing and PTGS in the Drosophila germline. Secondary ‘ping-pong’ piRNA processing is carried out by the cycling of target RNAs and piRNAs between the piwi-class endonucleases aubergine (aub) and argonaute 3 (AGO3) and this is intimately associated with destruction of mRNA target. (D) Post-transcriptional gene silencing mediated by mi/siRNAs can occur by directly interfering with translation. Thus, the mRNA level will remain unchanged, but the amount of protein product will be reduced.

The pathway of post-transcriptional gene silencing (PTGS) is largely dictated by the class of ncRNA and its interaction with the mRNA target within a specific RISC or RISC-related complex. Thus, to describe these processes, ncRNA-class specific GO BP terms have either been newly minted or existing terms modified to fit a common pattern. The terms share a common parent term, ‘*regulatory ncRNA-mediated post-transcriptional gene silencing*’ (GO:0035194) (Figure 1A) and the names have been formulated following the template: xRNA-mediated post-transcriptional gene silencing and child terms: xRNA-mediated gene silencing by mRNA destabilization and xRNA-mediated gene silencing by inhibition of translation. Where a more specific process occurs, the appropriate terms have been added in the hierarchy. In some cases, experimental evidence allows curators to select more detailed child terms for the BP aspect. Gene silencing by mRNA destabilization can be mediated by siRNAs, miRNAs and piRNAs. Both siRNAs and miRNAs act as part of a RISC (a complex based on the Argonaute AGO sub-family), where they base-pair with target mRNAs (Figure 1B). Where near perfect complementarity exists between the target mRNA and si/miRNA, mRNA destabilization is initiated by the endoribonucleolytic activity of an AGO protein within the RISC. This is more commonly associated with gene silencing in plants [19,20]. In animals, si/miRNA-mediated mRNA destabilization is generally initiated by deadenylation [21,22]. Experiments that demonstrate that PTGS is a result of a decrease in the amount of target mRNA can be captured using the terms ‘*siRNA-mediated gene silencing by mRNA destabilization*’ (GO:0090625) or ‘*miRNA-mediated gene silencing by mRNA destabilization*’ (GO:0035195). Similar to si/miRNAs, piRNAs act in a complex with an Argonaute family protein, but from the PIWI sub-family. The main mechanism for PTGS is PIWI-mediated endonucleolytic cleavage of the target mRNA. The piRNAs class of ncRNA are most commonly associated with the silencing of transposable elements (TEs) in the germline [4] and, where the transcript is derived from a TE, then *‘piRNA-mediated retrotransposon silencing by mRNA destabilization*’ (GO:0141009) can be used. However, there are a number of examples of piRNAs acting to target non-TE mRNAs, such as Drosophila maternal mRNAs in the syncytial embryo [23] and mouse spermatid mRNAs during spermatogenesis [24], in these cases ‘*piRNA-mediated gene silencing by mRNA destabilization*’ (GO:0140991) is the appropriate term. The biogenesis of piRNAs may be coupled to PTGS, where the piRNA-directed cleavage of the target mRNA results in the creation of a secondary piRNA (Figure 1C) in a self-perpetuating loop known as ‘*secondary piRNA processing*’ (GO:0140965) or the ‘ping-pong cycle’ [25]. Thus, there may be substantial overlap between the components annotated to ‘*secondary piRNA processing*’ (GO:0140965) and ‘*piRNA-mediated gene silencing by mRNA destabilization*’ (GO:0140991).

PTGS by regulatory ncRNAs can also directly suppress translation by the disrupting translation factor binding or by inhibiting ribosome loading on mRNAs (Figure 1D). In this case, the level of the target mRNA is unaffected, while the level of the protein product decreases over time. This is more commonly mediated by miRNAs and is captured with the term ‘*miRNA-mediated gene silencing by inhibition of translation*’ (GO:0035278). While siRNAs rarely act at the level of translation, this has been observed in plants for virus-derived siRNA and stress-induced endogenous 22nt siRNAs via an AGO1-dependent process [26,27]. In instances such as this, the BP term ‘*siRNA-mediated gene silencing by inhibition of translation*’ (GO:0070549) should be used.

### Heterochromatin formation mediated by siRNAs and piRNAs

**Figure 2.**
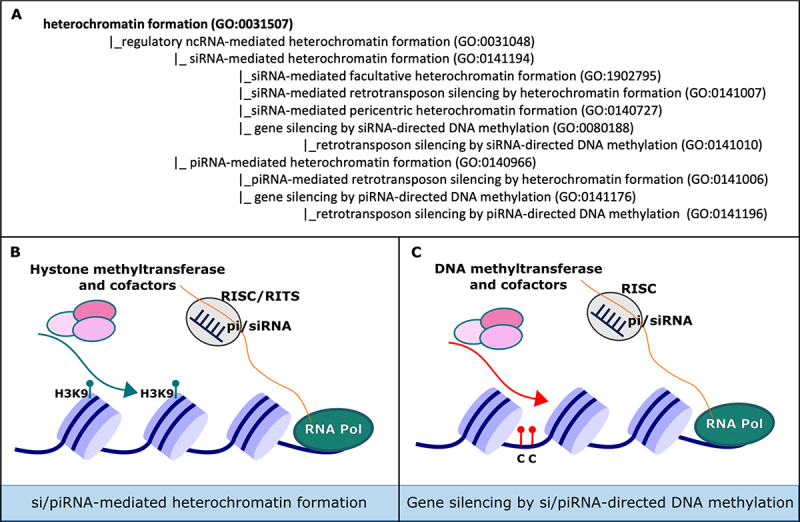
Regulatory ncRNAs can promote heterochromatin formation (A) illustration of the hierarchy of GO terms for the annotation of ncRNA-mediated heterochromatin formation. An expanded view provided in Figure S1C shows these terms in the context of the *‘regulation of gene expression’* (GO:0010468) branch of the GO. The formation of heterochromatin can be initiated by si/piRNAs by the recruitment of (B) histone modifiers or (C) DNA methyltransferases. This is dependent on the species and curators should be careful when selecting the term. Retrotransposon-specific processes are described by specific terms and are linked to the ‘*retrotransposon silencing*’ (GO:0010526) branch of the GO (not shown).

An important mechanism of gene silencing is through heterochromatin formation, whereby chromatin is compacted in a structure that is refractory to transcription. Both piRNAs and siRNAs have been shown to play an important role in inducing and maintaining heterochromatin. BP terms sitting in the branch ‘*regulatory ncRNA-mediated heterochromatin formation*’ (GO:0031048) were created or modified to describe the distinct nuclear processes mediated by regulatory ncRNAs (Figure 2A).

In the nucleus, mature regulatory ncRNAs incorporated in Argonaute family-containing complexes base-pair with nascent transcripts to direct the deposition of repressive marks at specific genomic loci [6]. The assembly of heterochromatin may be mediated via the recruitment of histone modifying complexes. For siRNAs, this process should be annotated to the BP term *‘siRNA-mediated heterochromatin formation’* (GO:0141194) or child terms (Figure 2B). For example, the term ‘*siRNA-dependent pericentric heterochromatin formation*’ (GO:0140727) can be used to annotate RNA-induced initiation of transcriptional silencing (RITS) complex components in *S. pombe*, which is required for the constitutive transcriptional silencing of centromeric regions [28,29]. Regulatory ncRNAs can also mediate transcriptional gene silencing by directing DNA methyltransferases at specific genomic loci, and resulting methylated CpG islands promote heterochromatin assembly at these regions [30] (Figure 2B). In plants, this process is mediated by siRNAs, annotated by the term *‘gene silencing by siRNA-directed DNA methylation’* (GO:0080188) (Figure 2C). In other species, piRNAs may direct *de novo* DNA-methylation, such as during spermatogenesis in mice, when *‘retrotransposon silencing by piRNA-directed DNA methylation’* (GO:0141196) is used to maintain genome integrity [31]. In *Drosophila*, which lacks an ortholog to mammalian *de novo* DNA methylase DNA methyltransferase 3, piRNA-PIWI complexes recruit histone modifying enzymes to TEs to direct *‘piRNA-mediated retrotransposon silencing by heterochromatin formation’* (GO:0141006).

### Annotating the functions of lncRNAs

LncRNAs are a very loosely defined class of ncRNAs; they are >200 nucleotides in length and may be transcribed from various genomic loci, including antisense, intronic, divergent and intergenic regions [7,32]. For GO curation, they present a particular challenge; the lack of primary sequence conservation or common motif or partner protein(s) means that functional classification based on RNA types, such as those made for miRNAs, piRNAs, and siRNAs, are difficult to apply. For lncRNAs, interpretation of function must be made on a case-by-case basis, more similar to the annotation of proteins than like other regulatory ncRNAs. Below we discuss examples of lncRNAs which we have reviewed and annotated to provide a template for the MF and BP terms for curators to employ to capture these diverse activities.

**Figure 3.**
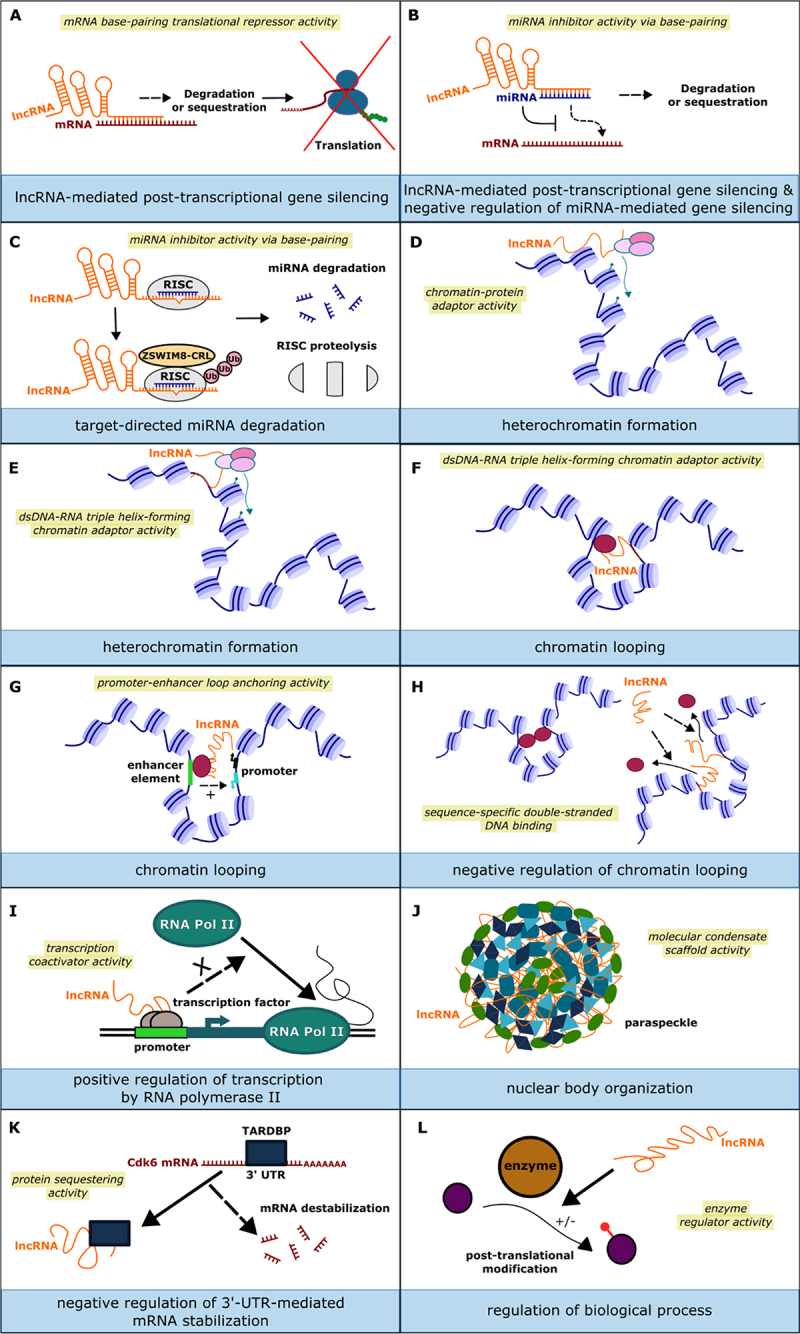
The diversity of lncRNA activities. Each panel depicts an activity that has been described for a lncRNA to accompany examples in the main text. The recommended MF term (or parent term) is highlighted in yellow and the BP term (or parent term) is shown in the bottom blue strip. LncRNAs can act post-transcriptionally to down-regulate mRNAs (A) or miRNAs (B) in the cytosol. (C) Target-directed miRNA degradation involves the RISC. It is not limited to lncRNAs and should therefore be co-annotated with ‘*lncRNA-mediated post-transcriptional gene silencing*’ (GO:0000512). (D-G) in the nucleus, lncRNAs can act as adaptor molecules to bring components together to influence chromatin structure. Some lncRNAs bind genomic DNA to recruit proteins (E,F) or to displace DNA-binding proteins (H). Other types of ‘*molecular adaptor activity*’ (GO:0060090) performed by lncRNAs include acting as transcriptional co-regulators (I) and assembling non-membrane bound organelles (J). LncRNAs have also been shown to sequester proteins, such as with the binding of TARDBP by the lncRNA Gadd7 (K) or inhibit enzymes (L). In the examples shown in this figure, many MFs can be annotated using terms from the ‘*molecular adaptor activity*’ (GO:0060090) branch of the GO and take part in BPs from the *‘cellular component organization’* (GO:0016043) branch. These terms are shown in the GO hierarchy in Figure S1B and Figure S1D, respectively.

Some lncRNAs have been shown to act in PTGS, for which the BP term ‘*lncRNA-mediated post-transcriptional gene silencing*’ (GO:0000512) was created. (Figures 1A, 3A,B). To date, more specific child terms have not been created as discrete, definable pathways are not apparent. The mechanism of PTGS occurs via their ability to base-pair with a target RNA, therefore co-annotation of lncRNAs with the MF terms ‘*mRNA base-pairing translational repressor activity*’ (GO:1903231) or *‘miRNA inhibitor activity via base-pairing’* (GO:0140869) can be used to point to the class of RNA that is the target of regulation, as well as extending the annotation with ‘*has_input*’ and the target ID. When lncRNAs base-pair with miRNAs, down-regulating miRNA interaction with bona fide mRNA targets, they are sometimes referred to as miRNA sponges, decoys or competing endogenous RNAs (ceRNAs) [33] and should also be annotated using the BP term ‘*negative regulation of miRNA-mediated gene silencing*’ (GO:0060965) (Figure 3B).

There is also an emerging, distinct and definable pathway of miRNA destruction, termed ‘target-directed microRNA degradation’ (TDMD), that a few lncRNAs, such as Drosophila lncRNA:marge [34], mouse Opa interacting protein 5, opposite strand 1 (Cyrano) [35] and human OIP5 antisense RNA 1 [36], have been shown to direct (Figure 3C). However, it should be noted that TDMD is not exclusively orchestrated by lncRNAs and there are examples of endogenous mRNAs and viral RNAs acting as triggers [37]. TDMD occurs when a miRNA is targeted for degradation by another complementary RNA and is gaining interest as a mechanism by which cells can rapidly shutdown gene silencing by specific miRNAs [34,36]. It occurs when the complementarity between miRNA and ‘target’ RNA in the RISC is more extensive, disrupting the interaction between AGO and the 3’ end of the miRNA, leading to ubiquitin-mediated degradation of AGO and/or miRNA tailing and trimming [35]. In either case, the instability of the RISC results in the complete degradation of the miRNA by cellular nucleases. Importantly, and distinguishing it from other RNA-mediated miRNA destruction pathways, the levels of the complementary RNA remain unchanged, allowing it to take part in further rounds of TDMD. As TDMD is initiated by RNA–RNA base-pairing, the term *‘miRNA inhibitor activity via base-pairing’* (GO:0140869) captures the mechanism by which RNAs can target miRNAs and the BP term ‘*target-directed miRNA degradation*’ (GO:0140958) was created to annotate the targeting RNA and other components of the pathway, such as ribonucleases and E3 ligases, that contribute to this process.

Due to their sequence and three-dimensional complexity, lncRNAs have the potential to bridge multiple interactions, acting as molecular scaffolds for DNA, RNA and protein complexes. When lncRNAs act to bring molecules together, curators use MF terms from the ‘*molecular adaptor activity*’ (GO:0060090) branch of GO (Figure S1B) and specify the molecules bound in the ‘*has_input*’ field. For lncRNAs that localize to chromatin and recruit factors that promote chromatin compaction, the MF term *‘chromatin-protein adaptor activity’* (GO:0140463) is appropriate, coupled with the BP terms in the ‘*heterochromatin formation’* (GO:0031507) branch (Figure 3D). A well-known example of a lncRNA with such an activity is mammalian Xist (X-inactive specific transcript), an essential component in mediating the silencing of one female X chromosome [38]. Transcribed from the inactive X chromosome, Xist acts as a molecular platform, recruiting and organizing effectors of gene silencing, such as the polycomb repressive complex 2 (PRC2), to chromatin. As such, its molecular function can be captured by annotation with *‘chromatin-protein adaptor activity’* (GO:0140463) involved in the process of *‘random inactivation of X chromosome’* (GO:0060816) which lies under the ‘*heterochromatin formation*’ (GO:0031507) branch. LncRNAs such as Fendrr, HOTAIR and MEG3 can act as sequence-specific DNA adaptors, bringing specific DNA loci and proteins in proximity to influence gene expression. This is mediated by the non-Watson – Crick base-pairing of the lncRNA to form a DNA:DNA:RNA triplex and lncRNA-protein interactions [39], described by the term ‘*dsDNA-RNA triple helix-forming chromatin adaptor activity*’ (GO:0141180) (Figure 3D). This may result in the recruitment of chromatin modifying proteins to specific loci, e.g., to promote ‘*heterochromatin formation’* (GO:0031507) (Figure 3E), or to be used to mediate longer-range interactions, such as ‘*chromatin looping*’ (GO:0140588) (Figure 3F). In mouse, the lncRNA encoded by *Hm629797* (also known as *Mrhl*) can mediate these activities, forming a DNA:DNA:RNA triplex with the *Sox8* promoter to bring it into proximity with a silencer element via interactions with cohesin and CCCTC-binding factor (CTCF) [40]. Hm629797 also recruits the PRC2 complex to deposit H3K27me3 repressive histone marks and promote the formation of heterochromatin to further silence the *Sox8* gene [40].

LncRNAs can promote gene expression by modifying chromatin architecture in other ways. For example, the lncRNA CASC11 (cancer susceptibility 11 or MYMLR) transcribed divergently from proto-oncogene *MYC*, enhances the transcription of *MYC* by promoting long-range promoter-enhancer interactions in concert with Poly(rC)-binding protein 2 [41]. The activities of CASC11 have been annotated with the MF terms ’*promoter-enhancer loop anchoring activity*’ (GO:0140585) (Figure 3G) and ‘*chromatin-protein adaptor activity*’ (GO:0140463) and the process annotated with ‘*chromatin looping*’ (GO:0140588) involved in the ‘*positive regulation of transcription by RNA polymerase II*’ (GO:0045944). As well as facilitating chromatin looping, lncRNAs may also inhibit it. The human lncRNA Jpx binds at thousands of genomic loci, competing with CTCF to prevent excessive chromatin looping and the inappropriate repression of numerous genes [42]. As this activity is via direct DNA binding, Jpx has been annotated with the MF term ‘*sequence-specific double-stranded DNA binding*’ (GO:1990837), involved in the ‘*positive regulation of transcription by RNA polymerase II*’ (GO:0045944) by ‘*negative regulation of chromatin looping*’ (GO:0160164) (Figure 3H).

As well as facilitating long-range chromatin structure, lncRNAs can also act as transcription factor co-regulators. An example of this is the Drosophila lncRNA:CR33942, which displays ‘*transcription coactivator activity*’ (GO:0003713) for the NFκB transcription factor Relish [43] to enhance the transcription of antimicrobial peptide genes via the ‘*positive regulation of transcription by RNA polymerase II*’ (GO:0045944). LncRNAs may also act to sequester transcription factors or mRNA binding proteins to influence gene expression. In CHO‐K1 cells, the lncRNA Gadd7 binds to and sequesters TAR-DNA binding protein (TARDBP), preventing it from interacting with the 3’UTR of Cdk6 mRNA and preventing its turn-over [44]. This activity was captured using the MF term *‘protein sequestering activity’* (GO:0140311) as part of the BP *‘negative regulation of 3’-UTR-mediated mRNA stabilization’* (GO:1905869) (Figure 3K).

Although lncRNAs are best known for their roles in controlling gene expression, there are other examples which illustrate that lncRNAs are as functionally divergent as their protein counterparts. The mammalian lncRNA NEAT1 mediates liquid–liquid phase separation of proteins in the assembly of paraspeckles [45]. This phase separation-promoting adaptor activity can be described using the MF term ‘*molecular condensate scaffold activity*’ (GO:0140693) and the BP term ‘*nuclear body organization’* (GO:0030575) (Figure 3J). LncRNAs have also been shown to regulate the post-translational modification of proteins. When the lncRNA directly targets the enzymatic activity of the modifier, then a MF term from the *‘enzyme regulator activity’* (GO:0030234) MF branch is used. This regulatory function is usually involved in *‘regulation of [a] biological process’* (GO:0050789) (Figure 3L). An example of this is human lnc-DC that binds to STAT3 (‘*STAT family protein binding*’ (GO:0097677)) and inhibits its dephosphorylation by PTPN6 (‘*protein phosphatase inhibitor activity*’ (GO:0004864)), promoting the translocation of STAT3 into the nucleus to activate transcription (‘*positive regulation of receptor signaling pathway via JAK-STAT*’ (GO:0046427) [46]. In *Drosophila*, lncRNA VSR interacting RNA (lncRNA:Vinr) functions in a yet uncharacterized antiviral pathway to promote the expression of antimicrobial peptides (AMPs), by binding to Cactin (‘*protein binding*’ (GO:0005515)) and preventing ubiquitination and destruction by the *‘negative regulation of proteasomal ubiquitin-dependent protein catabolic process’* (GO:0032435) resulting in the *‘positive regulation of antimicrobial humoral response’* (GO:0002760).

Thus, the roles of lncRNAs are far more diverse than those of the other regulatory ncRNAs. This survey validates our approach of annotating them in a similar fashion to that of proteins, with exceptions in cases where the MF mechanism is via base-pairing with an RNA target.

### GO annotation and networks analysis of ncRNAs

We have applied these new and revised terms to annotate examples from the research literature, and members of the GO Consortium (GOC) have assisted in the revision of existing annotations where required. However, these guidelines principally serve as a template to facilitate the expansion of annotation data for regulatory ncRNAs in the GO database from the many different contributors in the GOC. To this end, manuals for the annotation of lncRNAs, siRNAs and piRNAs have been provided for curators, and the miRNA manual has been updated (see Table S2 for resources).

To date, the number of manual annotations to BP terms under the ‘r*egulatory ncRNA-mediated gene silencing*’ (GO:0031047) branch of GO comprises 7,558 annotations to 4,075 distinct gene products (of which 2,759 annotations are to 825 distinct ncRNAs) from 1,937 publications (Table S4 gives a breakdown of annotations to specific terms). For base-pairing specific terms in the MF domain, ‘*mRNA base-pairing translational repressor activity*’ (GO:1903231), is the most frequently used as it has been in existence since 2014 (originally named *‘mRNA binding involved in posttranscriptional gene silencing’*) and its use was facilitated by the guidelines published by Huntley et al. [18]. Querying QuickGO (https://www.ebi.ac.uk/QuickGO/), 2,208 annotations have been made to this term, for 647 ncRNAs from experimental work in 1,306 research papers. For the majority of these annotations (2,199), targets have also been captured using the ‘*has_input*’ extension. The newly created terms: ‘*miRNA inhibitor activity via base-pairing*’ (GO:0140869) and ‘*dsDNA-RNA triple helix-forming chromatin adaptor activity*’ (GO:0141180) have been used to make 30 and 9 annotations, respectively. As highlighted in the introduction, in 2022, there were only 38 annotations associated with human lncRNAs, these have now increased to 214 and many as a direct result of our focused annotation of this class of ncRNAs.

**Figure 4.**
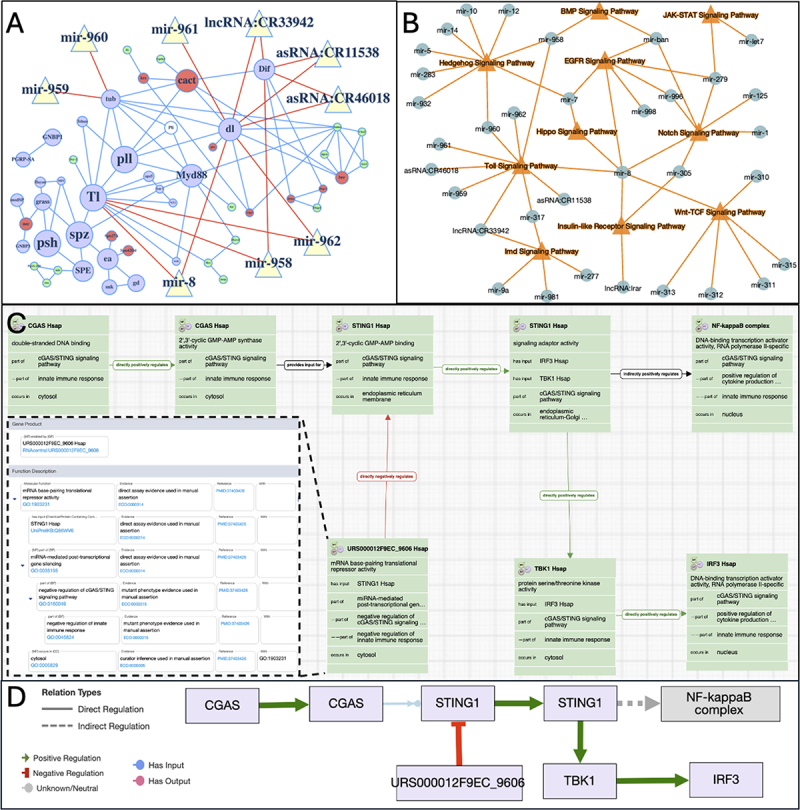
Modeling ncRNA interactions with signalling pathways using GO data. (A) A cytoscape.Js rendering of the FlyBase Drosophila Toll Signaling Pathway (FBgg0001059) network. ‘Core’ pathway components are shown in mauve, positive regulators in green, negative regulators in red, context-dependent regulators in white. The size of circular nodes (protein coding genes) is determined by the experimental data supporting pathway assignment [62]. The edges between nodes derived from physical interaction data. Regulatory ncRNAs are depicted by cream triangles (of uniform size, unrelated to support) and red lines connect them to their targets. (B) Intersection of ncRNAs with pathways in *Drosophila* based on pathway data curated by FlyBase using the GO. Signaling pathways are shown by orange triangles and ncRNAs by green circles. Multiple regulatory ncRNAs have been experimentally shown to interact and regulate each pathway. Some ncRNAs can target multiple pathways. (C) ‘Model of MiR-4691-3p inhibition of cGAS-sting signaling in the cytosol (human)’ gomodel:654d809000000802 in the noctua visual pathway Editor curation interface, showing the intersection of miR-4691-3p (RNAcentral:URS000012F9EC_9606) with the cGAS-sting pathway by the post-transcriptional gene silencing of STING1. The expanded box section shows the detailed annotations and evidence that is associated with the miR-4691-3p. (D) A simplified view of the GO-CAM shown in panel C rendered in cytoscape can be used to present the pathway constructed in the noctua curation interface. Currently these models can be viewed in AmiGO (https://amigo.geneontology.org/amigo/model/654d809000000802) and on Alliance gene page pathway tab (e.g. https://www.alliancegenome.org/gene/HGNC:21367#pathways).

There is an increasing understanding of the biomedical importance of ncRNAs in the development of various diseases [47–49], potential for use in therapies [50,51] and as biomarkers for disease [52,53]. Thus, it is imperative that, beyond capturing the mechanisms of action, the ncRNA targets and the impact of the ncRNAs on downstream cellular or system-level processes are captured. First, the targets of ncRNAs can be recorded by extending the annotation with the *‘has_input’* extension or curating pairwise physical interactions [54]. Second, systems-level impacts can be recorded by assigning broader, contextual BP GO terms to ncRNA. There are a number of resources that display and utilize this information (many summarized in [55]), for example, the GO annotation and ontology search tools AmiGO and QuickGO [56], the RNAcentral database which aggregates ncRNA data [57], the PSICQUIC molecular interactions server [58] and RNAenrich [59], a gene set enrichment analysis tool for ncRNAs and their targets. Published examples where such annotation information has been used to build complex models include the role of miR-21 in the epithelial-to-mesenchymal transition [60] and of the involvement of miRNAs in processes related to Alzheimer’s disease and neuroinflammatory processes [61]. We looked at an example of the integration of ncRNA GO annotation data in the FlyBase Signaling Pathway resource [62], where gene products with experimentally evidenced GO annotations to the regulation signalling pathways are used to populate pathway pages. An interaction network for the Toll Signalling pathway (Figure 4A), constructed from combining pairwise physical interaction data with pathway regulation annotation in Cytoscape.js interaction networks [63,64], shows that the lncRNAs asRNA:CR11538, asRNA:CR46018, and lncRNA:CR33942, act on the NFκB class transcription factors encoded by *Dif* and *dl* in the ‘*Toll signaling pathway*’ (GO:0008063). However, these lncRNAs have different regulatory roles: asRNA:CR11538 is involved in the ‘*negative regulation of Toll signaling pathway*’ (GO:0045751), acting with ‘*protein sequestering activity*’ (GO:0140311) on Dif and Dl, whilst asRNA:CR46018 [65] and lncRNA:CR33942 [64] are involved in the ‘*positive regulation of Toll signaling pathway*’ (GO:0045752) via ‘*NF-kappaB binding*’ (GO:0051059). The network shows that NFκB proteins of the Toll pathway are also targeted by a number of miRNAs: miR-8, miR-962, miR-961 and miR-958, which act via ‘*mRNA base-pairing translational repressor activity*’ (GO:1903231) in the ‘*negative regulation of Toll signaling pathway*’ (GO:0045751). This example shows how using the GO annotation to define the regulatory effect of the ncRNA on a process, i.e., positive or negative, gives a more nuanced view beyond just recording an interaction. Using the gene set analysis tool PANGEA [66] that integrates this information, we were able to visualize the regulation of Drosophila signalling pathways by ncRNAs (Figure 4B). In some instances regulatory ncRNAs intersect with multiple pathways, such as lncRNA:CR33942, which interacts with the Toll [64] and Imd (peptidoglycan recognition protein) signalling pathways [43]; and mir-8 interacts with 6 pathways, up-regulating Wnt-TCF (canonical Wnt), EGFR, Toll and Notch pathways, and down-regulating Hippo and Insulin-like Receptor pathways (Table 1). As Table 1 and Figure 4A show, ncRNAs often target more than one component of a pathway in the regulation of a process.

**Table 1.** Pathway GO annotations and targets of Drosophila lncRNA:CR33942 and mir-8. LncRNA:CR33942 and mir-8 can interact with and regulate multiple signalling pathways and target multiple components in a pathway (Figure 4B). The mechanism of action can be captured using GO terms from the MF aspect (column 1), the pathway regulation GO terms from the BP aspect (column 2). The targets and their roles are shown in column 3 and the reference (PMID) in column 4.

ncRNA gene|mechanism	Pathway GO term (ID)	Target gene|role	Ref
**lncRNA:CR33942**|NF-kappaB binding (GO:0051059)	positive regulation of Toll signalling pathway (GO:0045752)	**Dif**|transcription factor **dl**|transcription factor	[64]
**lncRNA:CR33942**|transcription coactivator activity (GO:0003713)	positive regulation of peptidoglycan recognition protein signalling pathway (GO:0061059)	**Rel**|transcription factor	[43]
**mir-8**|mRNA base-pairing translational repressor activity (GO:1903231)	negative regulation of Toll signalling pathway (GO:0045751)	**Tl**|receptor **dl**|transcription factor	[67]
**mir-8**|mRNA base-pairing translational repressor activity (GO:1903231)	negative regulation of Notch signalling pathway (GO:0045746)	**Ser**|receptor ligand	[68]
**mir-8**|mRNA base-pairing translational repressor activity (GO:1903231)	negative regulation of epidermal growth factor receptor signalling pathway (GO:0042059)	**spi**|receptor ligand	[69]
**mir-8**|mRNA base-pairing translational repressor activity (GO:1903231)	negative regulation of canonical Wnt signalling pathway (GO:0090090)	**wls**|ligand biogenesis **pan**|transcription factor	[70]
**mir-8**|mRNA base-pairing translational repressor activity (GO:1903231)	positive regulation of insulin receptor signalling pathway (GO:0046628)	**ush**|transcription factor	[71]
**mir-8**|mRNA base-pairing translational repressor activity (GO:1903231)	positive regulation of hippo signalling (GO:0035332)	**yki**|transcription factor **sd**|transcription factor	[72]

To enhance the systems-level potential of GO annotation, the GOC has developed a framework termed GO Causal Activity Modeling (GO-CAM; [73], which allows GO annotations to be linked in a defined schema to make larger, semantically structured pathway models. We employed GO-CAM to illustrate how units of information about human miR-4691-3p (RNAcentral:URS000012F9EC_9606) can be integrated into a larger pathway model of the innate immune system cGAS/STING signalling. The information: miR-4691-3p acts via its ‘*mRNA base-pairing translational repressor activity*’ (GO:1903231) as part of ‘*miRNA-mediated post-transcriptional gene silencing*’ (GO:0035195) of STING1 occurring in the *‘cytosol’* (GO:0005829) as part of *‘negative regulation of cGAS/STING signaling pathway’* (GO:0160049), as part of the *‘negative regulation of innate immune response*’ (GO:0045824) was curated from [74] and integrated into a GO-CAM of the human cGAS/STING signalling pathway. Following the relational links, we can infer that the activity of miR-4691-3p will negatively impact the transcription factor activity of the NF-kappaB complex and IRF3, thus capturing the downstream causal effects. Although GO-CAMs are still relatively new, as they grow as a resource, they promise to allow the ability to infer consequences of regulation across complex networks. Furthermore, they can be ‘decomposed’ to standard GO annotations, so can serve a more conventional role as well [73] and so can be integrated into standard curation work flows and data analysis by downstream users.

## Discussion

The discovery of RNA interference in 1998 in *C. elegans* has arguably seeded the study of regulatory ncRNAs as a field of research (for a comprehensive history see [75]. Due to advances in sequencing technology [76], the number of catalogued ncRNA sequences has increased rapidly. RNAcentral, a cross-species integrative database, houses over 35 million ncRNA sequences (rnacentral.org, release 24). The relative youth of this field of study, coupled with the large and increasing number of sequenced, potentially regulatory, ncRNAs, means that the systemization of functional knowledge of ncRNAs lags far behind that of protein-coding genes. As more becomes known about the nature and diversity of ncRNAs, their molecular mechanisms and the processes they contribute to, there is a need to match this with provision of expanded bioinformatics resources.

The expansion and revision of the GO presented in this paper is aimed at supporting the improvement of ncRNA annotation, both in terms of classification of their molecular mechanisms of action and the biological route via which they attain a regulatory outcome. This will facilitate the translation of observations from research publications into systematic, computationally accessible knowledge. As many high-throughput analysis tools incorporate GO annotations, more informative ncRNA annotations will contribute to improved data interpretation. Additionally, this work has direct impact on computational GO term assignment such as those made by Rfam, a database of manually curated ncRNA families [77], which associates GO terms to Rfam classes. These GO terms can be propagated to ncRNAs via their Rfam assignment [57] and marked with the GO evidence code IEA (Inferred from Electronic Annotation) and the Rfam ID to allow users to trace provenance. This is an important mechanism for the annotation of ncRNAs that have not yet been the focus of much research. Looking to the future, artificial intelligence tools might become increasingly useful for ncRNA classification purposes; however, their training will rely on the existence of sufficient amounts of accurately curated information. Providing the framework for this is a prerequisite to fully exploit such technology.

Although this manuscript may be of primary interest to GO curators, it is important that researchers understand how the narrative in research publications is translated into systemized annotations. We have therefore explicitly exposed the identifiers (IDs) we use in curation (see main text, Table S1 and S3). These are important – they allow the tracking of information even if names/entities/controlled vocabulary terms change or are deprecated and enable the flow of information between bioinformatics resources. With ncRNAs, because the field is relatively young and the expansion of sequence information is so rapid, it is sometimes difficult to map ncRNAs named in papers to persistent identifiers, which are required for GO curation. Much of a curator’s time can be spent tracking down which ncRNAs were studied in papers and in some cases it is not possible to curate the data because there is not enough information to accurately identify the ncRNA. A common time-sink is having to manually extract sequence information embedded in figures of research articles. For example, it is common for papers to only include miRNA sequences in diagram that shows the mismatch between an mRNA target and a guide miRNA, with no information as to whether the miRNA is a −5p or −3p species and, as an added challenge to the curator, shown in a 3’-5’ orientation. This necessitates the curator typing out the sequence (a potential source of errors) to enable a search for the correct ID. Although, since the introduction of FAIR Data Principles [78] and compliance efforts by journals, we have seen much improvement, as curators we would encourage the inclusion of IDs (e.g. species gene IDs, GenBank accession and RNAcentral IDs) and sequences (in an accessible form, linear, 5’-3’) – which, if novel, should be deposited in the appropriate database. Examples of suitable IDs used in GO curation include organism specific gene identifiers, UniProtKB identifiers for proteins, or RNAcentral IDs for all classes of ncRNAs (see Table S3 for examples from this paper). By including these details, research funding can be more efficiently translated into usable data with a potential reach far beyond the initial publication.

The regulatory roles of ncRNAs are subject to very active research. As more sequence and functional characterization becomes available, there is an increased need for focused GO curation. This work will provide a springboard for such curation efforts and, with the integration of this data in various data and informatics resources, will contribute to the utility of such data in building complex network models. Similar to the annotation of miRNAs reported here, the introduction of GO terms for other classes of ncRNAs now means that progress can also be made to systematically curate the functionality of a broader range of ncRNAs.

## Materials and methods

### GO annotation and review

Annotations for this article refer to standard GO annotations as described in The Gene Ontology Consortium 2023^81^. The curation tool Protein2GO (EMBL-EBI [79]; has been used to revise and add annotations. Annotations have been reviewed using AmiGO and QuickGO [56] to search and download annotations. The review process has been managed using the annotation section of the GO Consortium GitHub repository (https://github.com/geneontology/go-annotation).

### Ontology editing

Revision of the GO has been performed using the ontology editor Protege-5.6.1 (https://github.com/protegeproject [80] and the workflow managed in the ontology section of the GO Consortium GitHub repository (https://github.com/geneontology/go-ontology). Alongside adding new terms to the ontology, as well as reviewing and updating existing ones, incorrect or redundant terms have been obsoleted, so they can’t be used for annotation in the future. A list of changes (new terms, updated terms, and obsoletions) can be found in Table S1.

### Release information and metrics

The data used for analysis was downloaded from QuickGO (GO version 2024-06-13; Annotation set created on 2024-06-13 06:09). The number of GO annotations were obtained using the QuickGO annotations browser (https://www.ebi.ac.uk/QuickGO/annotations) statistics download and filtering by: GO term: GO:0031047 r*egulatory ncRNA-mediated gene silencing*, relations: is_a, part_of, occurs_in, regulates; Aspect: Biological Process; Evidence: ECO:0000352 (*evidence used in manual assertion*) and for ncRNAs only, Gene Product: RNA. MF annotations were filtered using the specific GO term and Evidence: ECO:0000352 (*evidence used in manual assertion*). The number of manual annotations for human miRNAs and lncRNAs were obtained from the human ncRNA annotation file (goa_human_rna.gaf.gz; dated 2024-06-14) downloaded from https://ftp.ebi.ac.uk/pub/databases/GO/goa/HUMAN/).

### PANGEA analysis

A Drosophila ncRNA gene set was generated by using the FlyBase vocabularies tool (http://flybase.org/vocabularies), querying with the Sequence Ontology term ‘*ncRNA_gene*’ (SO:0001263) to generate a hitlist of 3,605 genes. This list was exported to the PANGEA Fly page (https://www.flyrnai.org/tools/pangea/web/home/7227) using a link in the drop-down ‘Export’ menu. The gene set selected was ‘FlyBase signaling pathway (experimental evidence)’. The results were visualized by selecting the groups shown in Figure 4B for display in the ‘Gene Set Node Graph’.

### GO-CAM

The ‘Model of MiR-4691-3p inhibition of cGAS-STING signalling in the cytosol (Human)’ gomodel:654d809000000802 was constructed in Noctua (http://noctua.geneontology.org) using the Visual Pathway Editor tool.

## Supplementary Material

Supplementary_materials_revised.docx

## Data Availability

The data that support the findings of this study are available in figshare at https://doi.org/10.6084/m9.figshare.c.7355434.v1. These data were derived from the following resources available in the public domain: QuickGO https://www.ebi.ac.uk/QuickGO/annotations, and GOA https://ftp.ebi.ac.uk/pub/databases/GO/goa/HUMAN/.
