# Supplementary material for: Representation of non-coding RNA-mediated regulation of gene expression using the Gene Ontology
Source: RNA Biol. 2024 Oct 7;21(1):36–48. doi: 10.1080/15476286.2024.2408523 (PMC11459742; doi:10.1080/15476286.2024.2408523)
Supplement: Supplementary_materials_revised.docx [file KRNB_A_2408523_SM3826.docx]

**Supplementary materials**

**Supplementary figures**

**
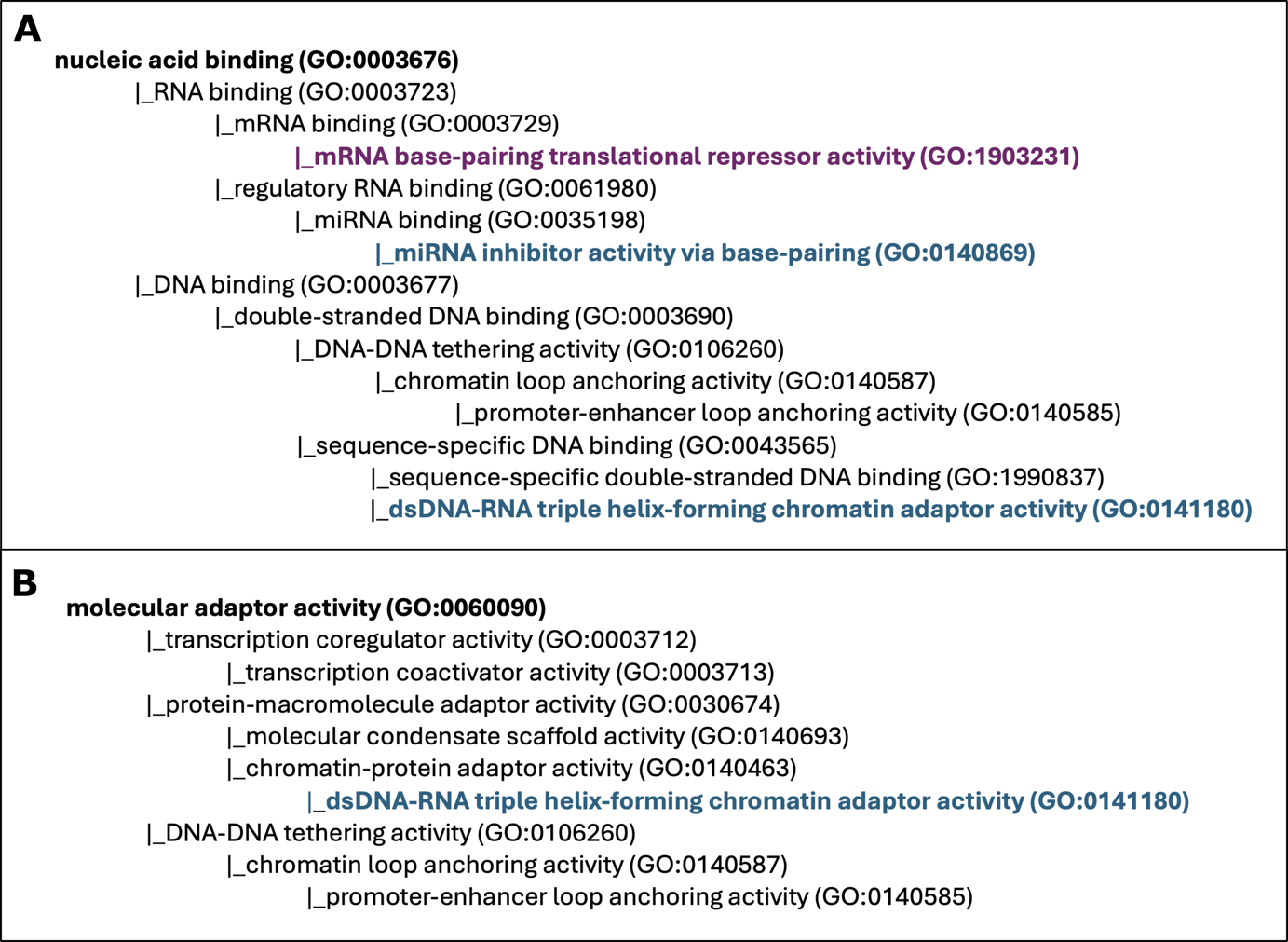
**

**
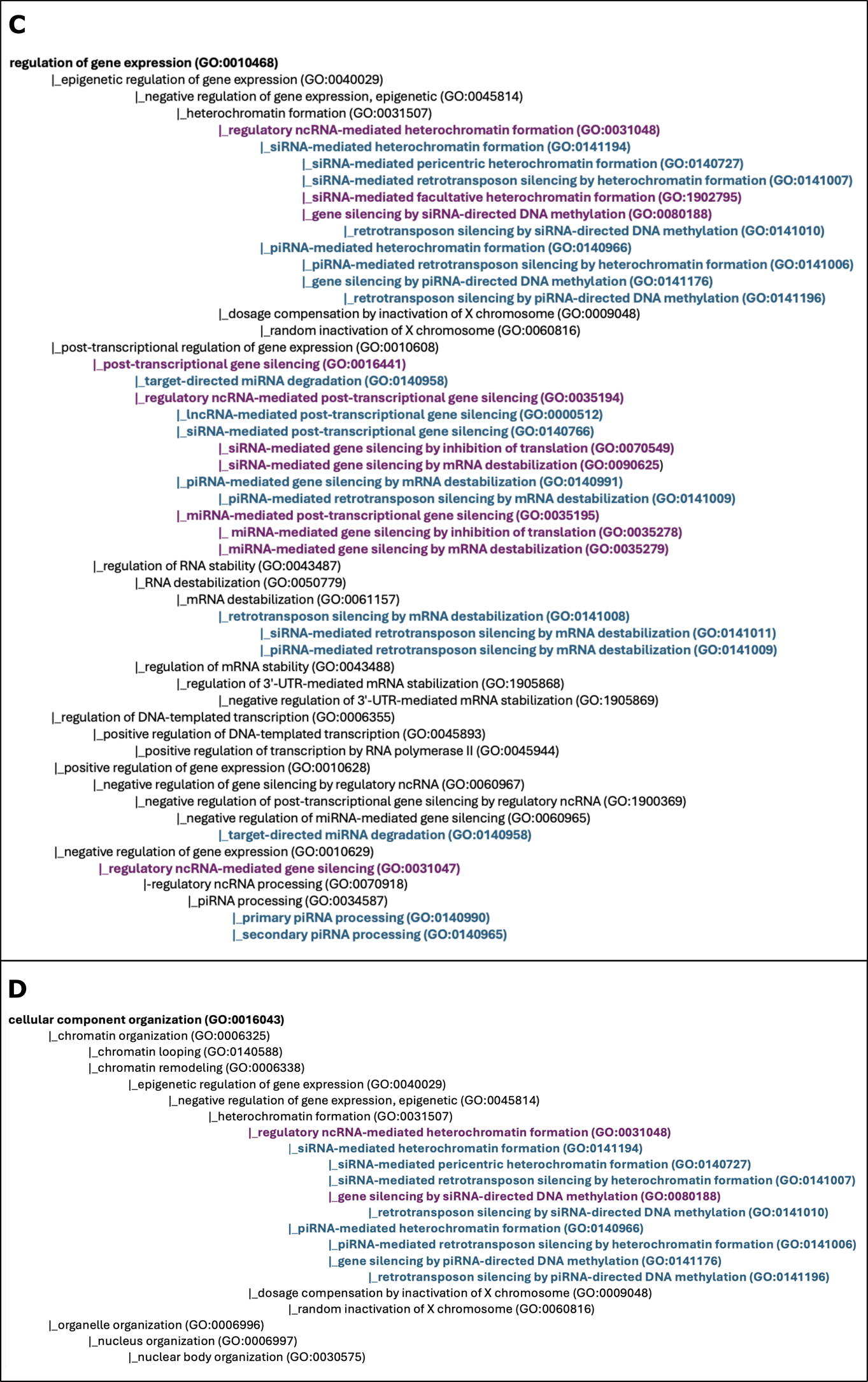
**

**Fig. S1 Hierarchical representation of the GO terms added or renamed in this paper. (A)** Illustration of the hierarchy of terms in **(A)** the *‘nucleic acid binding’* (GO:0003676), **(B)** the *‘molecular adaptor activity’* (GO:0060090), **(C)** the *‘regulation of gene expression’* (GO:0010468) and **(D)** the *‘cellular component organization’* (GO:0016043) branches of the GO. New terms are highlighted in blue and renamed terms are highlighted in purple, and correspond to terms listed in **Table S1**. The tree view displayed in this figure is simplified and does not show all possible paths. The ontological relation between nested terms are ‘*is_a’* (represented by |_), with the exception of the relation between ‘*regulatory ncRNA-mediated gene silencing’* (GO:0031047) and ‘*regulatory ncRNA processing’* (GO:0070918) in panel **C**, which is ‘*part_of*’ (represented by |-).

**Supplementary tables**

| **Aspect** | **GO ID** | **Name** | **Action** |
| --- | --- | --- | --- |
| MF | GO:1903231 | mRNA binding involved in post-transcriptional gene silencing | Renamed to mRNA base-pairing translational repressor activity |
| BP | GO:0016441 | posttranscriptional gene silencing | Renamed to post-transcriptional gene silencing |
| BP | GO:0031047 | RNA-mediated gene silencing | Renamed to regulatory ncRNA-mediated gene silencing |
| BP | GO:0035194 | post-transcriptional gene silencing by RNA | Renamed to regulatory ncRNA-mediated post-transcriptional gene silencing |
| BP | GO:0035195 | gene silencing by miRNA | Renamed to miRNA-mediated post-transcriptional gene silencing |
| BP | GO:0035279 | mRNA cleavage involved in gene silencing by miRNA | Renamed to miRNA-mediated gene silencing by mRNA destabilization |
| BP | GO:0070549 | negative regulation of translation involved in RNA interference | Renamed to siRNA-mediated gene silencing by inhibition of translation |
| BP | GO:0035278 | miRNA-mediated inhibition of translation | Renamed to miRNA-mediated gene silencing by inhibition of translation |
| BP | GO:0031048 | chromatin silencing by small RNA | Renamed to regulatory ncRNA-mediated heterochromatin formation |
| BP | GO:0090625 | mRNA cleavage involved in gene silencing by siRNA | Renamed to siRNA-mediated gene silencing by mRNA destabilization |
| BP | GO:0080188 | RNA-directed DNA methylation | Renamed to gene silencing by siRNA-directed DNA methylation |
| BP | GO:1902795 | heterochromatin domain assembly | Renamed to siRNA-mediated facultative heterochromatin formation |
| MF | GO:0140869 | miRNA inhibitor activity via base-pairing | new term |
| MF | GO:0141180 | dsDNA-RNA triple helix-forming chromatin adaptor activity | new term |
| BP | GO:0000512 | lncRNA-mediated post-transcriptional gene silencing | new term |
| BP | GO:0140990 | primary piRNA processing | new term |
| BP | GO:0140965 | secondary piRNA processing | new term |
| BP | GO:0140966 | piRNA-mediated heterochromatin formation | new term |
| BP | GO:0140991 | piRNA-mediated gene silencing by mRNA destabilization | new term |
| BP | GO:0141006 | piRNA-mediated retrotransposon silencing by heterochromatin formation | new term |
| BP | GO:0141007 | siRNA-mediated retrotransposon silencing by heterochromatin formation | new term |
| BP | GO:0141008 | retrotransposon silencing by mRNA destabilization | new term |
| BP | GO:0141009 | piRNA-mediated retrotransposon silencing by mRNA destabilization | new term |
| BP | GO:0141010 | retrotransposon silencing by RNA-directed DNA methylation | new term |
| BP | GO:0141011 | siRNA-mediated retrotransposon silencing by mRNA destabilization | new term |
| BP | GO:0140766 | siRNA-mediated post-transcriptional gene silencing | new term |
| BP | GO:0140727 | siRNA-dependent pericentric heterochromatin formation | new term |
| BP | GO:0141194 | siRNA-mediated heterochromatin formation | new term |
| BP | GO:0141176 | gene silencing by piRNA-directed DNA methylation | new term |
| BP | GO:0141196 | retrotransposon silencing by piRNA-directed DNA methylation | new term |
| BP | GO:0140958 | target-directed miRNA degradation | new term |
| BP | GO:0016458 | gene silencing | obsolete |
| BP | GO:0032199 | reverse transcription involved in RNA-mediated transposition | obsolete |
| BP | GO:0061587 | tRNA locus-associated negative regulation of gene expression | obsolete |
| BP | GO:0120085 | transposon integration involved in RNA-mediated transposition | obsolete |
| BP | GO:0150163 | miRNA-mediated activation of transcription by RNA polymerase II | obsolete |
| BP | GO:0150164 | miRNA-mediated regulation of transcription by RNA polymerase II | obsolete |
| BP | GO:0150165 | miRNA-mediated inhibition of transcription by RNA polymerase II | obsolete |
| BP | GO:0000335 | negative regulation of transposition, DNA-mediated | obsolete |
| BP | GO:0000336 | positive regulation of transposition, DNA-mediated | obsolete |
| BP | GO:0000337 | regulation of transposition, DNA-mediated | obsolete |
| BP | GO:0010525 | regulation of transposition, RNA-mediated | obsolete |
| BP | GO:0010527 | positive regulation of transposition, RNA-mediated | obsolete |
| BP | GO:0098806 | miRNA-mediated gene silencing by mRNA deadenylation | obsolete |
| BP | GO:0010528 | regulation of transposition | obsolete |
| BP | GO:0010529 | negative regulation of transposition | obsolete |
| BP | GO:0010530 | positive regulation of transposition | obsolete |
| BP | GO:0070928 | regulation of mRNA stability, ncRNA-mediated | obsolete |
|  |  |  |  |

**Table S1: Changes in the post-transcriptional gene silencing terms.** A summary of revisions to the GO to improve annotation accuracy for ncRNAs and the processes involving them. Where terms were obsoleted, genes were reviewed and reannotated to ensure that the biological roles were captured using the new guidelines and terms. Note that current rules for creating new GO terms require that the role has been experimentally validated. For a hierarchical view of the added and renamed terms, see **Fig. S1**.

| **Resources** | **Category** | **Website** |
| --- | --- | --- |
| QuickGO | GO annotations & Ontology | https://www.ebi.ac.uk/QuickGO |
| AmiGO | GO annotations & Ontology | https://amigo.geneontology.org/amigo |
| FlyBase Pathway Resource | GO annotations & Networks | http://flybase.org/lists/FBgg/pathways |
| Gene Ontology: Causal Activity Models | GO-CAMs | https://geneontology.cloud/browse |
| Noctua tool | GO-CAM curation | http://noctua.geneontology.org/workbench/noctua-landing-page |
| GO Introduction to GO-CAM | Overview | https://geneontology.org/docs/gocam-overview/ |
| GO Guide to GO-CAMs and Noctua | Curation guidelines | https://wiki.geneontology.org/index.php/Noctua |
| GO guide to lncRNAs | Curation guidelines | https://wiki.geneontology.org/LncRNA_GO_annotation_manual |
| GO guide to miRNAs | Curation guidelines | https://wiki.geneontology.org/MicroRNA_GO_annotation_manual |
| GO guide to siRNAs | Curation guidelines | https://wiki.geneontology.org/SiRNA_GO_annotation_manual |
| GO guide to piRNAs | Curation guidelines | https://wiki.geneontology.org/PiRNA_GO_annotation_manual |
| GO guide to ‘has_input’ | Curation guidelines | https://wiki.geneontology.org/Has_input |
| LncRNADisease v3.0 | LncRNA Disease annotations | http://www.rnanut.net/lncrnadisease/ |
| RNAenrich | Gene Set Enrichment Analysis for ncRNA targets | http://idrblab.cn/rnaenrich/ |
| PANGEA | Gene Set Enrichment Analysis | https://www.flyrnai.org/tools/pangea/ |
| PSICQUIC | Interactions | http://www.ebi.ac.uk/Tools/webservices/psicquic/view/home.xhtml |
| RNAcentral | Centralized ncRNA database | https://rnacentral.org |
| UniProt | Centralized protein database | https://www.uniprot.org/ |
| Mouse Genome Informatics (MGI) | Mouse Model Organism Database | https://www.informatics.jax.org/ |
| HUGO Gene Nomenclature Committee (HGNC) | Human Genes Database | https://www.genenames.org/ |
| FlyBase | Drosophila Model Organism Database | http://flybase.org/ |

**Table S2. Resources.** A list of resources named in the text.

| **Gene name** | **Gene symbol** | **DB:Gene ID** | **UniProtKB/RNAcentral ID** |
| --- | --- | --- | --- |
| HOX transcript antisense RNA | HOTAIR | HGNC:33510 | URS00026A23F2_9606 |
| MIR149 | microRNA 149 | HGNC:31536 | URS00001C770D_9606 (hsa-miR-149-5p) |
| MIR217 | microRNA 217 | HGNC:31594 | URS000041E210_9606  (hsa-miR-217-5p) |
| HNRNPA1 | heterogeneous nuclear ribonucleoprotein A1 | HGNC:31594 | P09651 |
| DACH1 | dachshund family transcription factor 1 | HGNC:2663 | Q9UI36 |
| Aub | Aubergine | FB:FBgn0000146 | O76922 |
| AGO3 | Argonaute 3 | FB:FBgn0250816 | Q7PLK0 |
| Hen1 | Hen1 methyltransferase | FB:FBgn0033686 | Q7K175 |
| Piwil2 | piwi-like RNA-mediated gene silencing 2 | MGI:MGI:1930036 | Q8CDG1 |
| Piwil4 | piwi-like RNA-mediated gene silencing 4 | MGI:MGI:3041167 | Q8CGT6 |
| Piwil1 | piwi-like RNA-mediated gene silencing 1 | MGI:MGI:1928897 | Q9JMB7 |
| lncRNA:marge | long non-coding RNA:marge | FB:FBgn0263380 | URS00000B0CEE_7227  URS00000037B7_7227 |
| Oip5os1 | Opa interacting protein 5 | MGI:MGI:1913852 | URS000075A502_10090UURS000075B8A8_10090 |
| OIP5-AS1 | OIP5 antisense RNA 1 | HGNC:43563 | URS0000CCE04C_9606 |
| Xist | inactive X specific transcripts | MGI:MGI:98974 | URS000077A8CE_10090 |
| Fendrr | Foxf1 adjacent non-coding developmental regulatory RNA | MGI:MGI:1916040 | URS00007743A8_10090 |
| MEG3 | maternally expressed 3 | HGNC:14575 | URS0000759EA9_9606 |
| Hm629797 | cDNA sequence HM629797 | MGI:MGI:5440479 | URS0000765E64_10090 |
| Sox8 | SRY (sex determining region Y)-box 8 | MGI:MGI:98370 | Q04886 |
| Ctcf | CCCTC-binding factor | MGI:MGI:109447 | Q61164 |
| CASC11 | cancer susceptibility 11 | HGNC:48939 | URS0000392533_9606 |
| PCBP2 | poly(rC) binding protein 2 | HGNC:8648 | Q15366 |
| JPX | JPX transcript, XIST activator | HGNC:37191 | URS000075CF99_9606 |
| CTCF | CCCTC-binding factor | HGNC:13723 | P49711 |
| long non-coding RNA:CR33942 | lncRNA:CR33942 | FB:FBgn0062961 | URS0000809E6D_7227 |
| Relish | Rel | FB:FBgn0014018 | Q94527 |
| gadd7 | Long non-coding RNA Gadd7 | ENA:HG975394.1:1..754:ncRNA | URS0000698F2B_10030 |
| Tardbp | TAR DNA-binding protein 43 | UniProtKB:A0A8C2LUF5 | A0A8C2LUF5 |
| Cdk6 | Cyclin dependent kinase 6 | UniProtKB:A0A8C2LII0 | A0A8C2LII0 |
| NEAT1 | nuclear paraspeckle assembly transcript 1 | HGNC:30815 | URS000075DAEC_9606 |
| HSALNT0243356 (lnc-DC)* | - | ENA:KJ020272.1:1..397:ncRNA | URS00007B42BD_9606 |
| STAT3 | Signal transducer and activator of transcription 3 | HGNC:11364 | P40763 |
| PTPN6 | Tyrosine-protein phosphatase non-receptor type 6 | HGNC:9658 | P29350 |
| lncRNA:Vinr | VSR interacting RNA | FB:FBgn0287633 | URS00019813B3_7227 |
| cactin | cactin | FB:FBgn0031114 | Q9VR99 |
| MIR21 | microRNA 21 | HGNC:31586 | URS000039ED8D_9606  (hsa-miR-21-5p) |
| asRNA:CR11538 | antisense RNA:CR11538 | FB:FBgn0017424 | URS00009D92B3_7227 |
| asRNA:CR46018 | antisense RNA:CR46018 | FB:FBgn0267682 | URS0000A1266B_7227 |
| mir-8 stem loop | mir-8 | FB:FBgn0262432 | URS0000562752_7227 |
| Dorsal-related immunity factor | Dif | FB:FBgn0011274 | P98149 |
| dorsal | dl | FB:FBgn0260632 | P15330 |
| mir-962 | mir-962 stem loop | FB:FBgn0262200 | URS0000114018_7227  (dme-miR-959-3p) |
| miR-961 | miR-961 stem loop | FB:FBgn0262239 | URS00005C8829_7227 (dme-miR-961-3p) |
| miR-958 | miR-958 stem loop | FB:FBgn0262249 | URS0000187D5B_7227  (dme-miR-958-3p) |
| Toll | Tl | FB:FBgn0262473 | P08953 |
| Serrate | Ser | FB:FBgn0004197 | P18168 |
| spitz | spi | FB:FBgn0005672 | Q01083 |
| wntless | wls | FB: FBgn0036141 | Q95ST2 |
| pangolin | pan | FB:FBgn0085432 | Q8IMA8 |
| u-shaped | ush | FB:FBgn0003963 | Q9VPQ6 |
| yorkie | yki | FB:FBgn0034970 | Q45VV3 |
| scalloped | sd | FB:FBgn0003345 | P30052 |
| MIR4691 | microRNA 4691 | HGNC:41796 | URS000012F9EC_9606  (hsa-miR-4691-3p) |
| STING1 | stimulator of interferon response cGAMP interactor 1 | HGNC:27962 | Q86WV6 |
| IRF3 | interferon regulatory factor 3 | HGNC:6118 | Q14653 |

**Table S3. Genes and gene products in text.** Table of genes mentioned in the text. The gene symbols and names are taken from the database from column 3. Database (DB) abbreviations: HGNC: HUGO Gene Nomenclature Committee FB: FlyBase, MGI: Mouse Genome Informatics, ENA: European Nucleotide Archive. The UniProtKB identifiers are Swiss-Prot/UniProtKB or part of the Gene Centric Reference Proteome for that organism. For miRNAs, the 3p or 5p species is identified using the RNAcentral ID and the RNAcentral symbol is listed beneath the ID. *Lnc-DC was identified by searching RNAcentral with sequence data in supplemental material by [(Wang et al. 2014)](https://www.zotero.org/google-docs/?2GADa0) as corresponding to URS00007B42BD_9606, LncBook: transcript HSALNT0243356.

| **GO ID** | **Name** | **# ANNOTATIONS** |
| --- | --- | --- |
| GO:0031047 | regulatory ncRNA-mediated gene silencing | 118 |
| GO:0035194 | regulatory ncRNA-mediated post-transcriptional gene silencing | 513 |
| GO:0035195 | miRNA-mediated post-transcriptional gene silencing | 2441 |
| GO:0035279 | miRNA-mediated gene silencing by mRNA destabilization | 267 |
| GO:0035278 | miRNA-mediated gene silencing by inhibition of translation | 387 |
| GO:0140766 | siRNA-mediated post-transcriptional gene silencing | 0* |
| GO:0070549 | siRNA-mediated gene silencing by inhibition of translation | 5 |
| GO:0090625 | siRNA-mediated gene silencing by mRNA destabilization | 26 |
| GO:0140991 | piRNA-mediated gene silencing by mRNA destabilization | 30 |
| GO:0141009 | piRNA-mediated retrotransposon silencing by mRNA destabilization | 4 |
| GO:0031048 | regulatory ncRNA-mediated heterochromatin formation | 336 |
| GO:0141194 | siRNA-mediated heterochromatin formation | 0* |
| GO:1902795 | siRNA-mediated facultative heterochromatin formation | 1 |
| GO:0141007 | siRNA-mediated retrotransposon silencing by heterochromatin formation | 22 |
| GO:0140727 | siRNA-mediated pericentric heterochromatin formation | 37 |
| GO:0080188 | gene silencing by siRNA-directed DNA methylation | 117 |
| GO:0141010 | retrotransposon silencing by siRNA-directed DNA methylation | 4 |
| GO:0140966 | piRNA-mediated heterochromatin formation | 1 |
| GO:0141006 | piRNA-mediated retrotransposon silencing by heterochromatin formation | 83 |
| GO:0141176 | gene silencing by piRNA-directed DNA methylation | 6 |
| GO:0141196 | retrotransposon silencing by piRNA-directed DNA methylation | 55 |

**Table S4. Manual annotations directly associated with regulatory ncRNA-mediated gene silencing terms.** The terms in this table are taken from Fig. 1 and Fig. 2. The number of manual annotations listed are direct to each term and do not take into account the GO hierarchy or ‘regulates’ relations in the GO. (* ‘*siRNA-mediated heterochromatin formation*’ and ‘*siRNA-mediated post-transcriptional gene silencing*’ have not been used directly in annotation, but their child terms have). The numbers were downloaded from QuickGO (GO version 2024-06-13; Annotation set created on 2024-06-13 06:09) with the selection: Aspect: Biological Process and Evidence: ECO:0000352 (*evidence used in manual assertion*).
